# Angiogenin Levels and Their Association with Cardiometabolic Indices Following Vitamin D Status Correction in Saudi Adults

**DOI:** 10.3390/biology11020286

**Published:** 2022-02-11

**Authors:** Ghadeer M. Aldawsari, Shaun Sabico, Abir A. Alamro, Amal Alenad, Kaiser Wani, Abdullah M. Alnaami, Malak N. K. Khattak, Mohammad S. Masoud, Nasser M. Al-Daghri, Majed S. Alokail

**Affiliations:** 1 Biochemistry Department, College of Science, King Saud University, Riyadh 11451, Saudi Arabia; ghadeer.majed@gmail.com (G.M.A.); ssabico@ksu.edu.sa (S.S.); aalamro@ksu.edu.sa (A.A.A.); aalenad@ksu.edu.sa (A.A.); malokail@ksu.edu.sa (M.S.A.); 2 Chair for Biomarkers of Chronic Diseases, College of Science, King Saud University, Riyadh 11451, Saudi Arabia; kwani@ksu.edu.sa (K.W.); aalnaami@ksu.edu.sa (A.M.A.); mkhattak@ksu.edu.sa (M.N.K.K.); mohammad_masoud@hotmail.com (M.S.M.)

**Keywords:** vitamin D supplementation, angiogenin, apolipoproteins, cardiometabolic indices, HDL-cholesterol

## Abstract

**Simple Summary:**

Angiogenin (ANG) is a small 123 amino acid protein which in normal growth is associated with formation of new blood vessels in a process called angiogenesis; however, the abnormal levels of this protein in blood has been associated with diseases such as cancer, neurological disorders, and cardiovascular diseases. Vitamin D deficiency and elevated levels of blood lipids have also been associated with many diseases including cardiovascular diseases. In this study, the investigators tried to evaluate the relationship between the circulating levels of ANG, vitamin D, and lipids. The model used was vitamin D supplementation of deficient Saudi adults in order to find the effect of vitamin D correction on circulating levels of ANG and blood lipids. With vitamin D supplementation, modest but non-significant elevation in ANG was observed, as well as significant increase in apolipoproteins CIII and E and significant decrease in apo B. In addition, the correlation between circulating levels of ANG and apolipoproteins especially apo E observed in this study are interesting and should be investigated more as both are linked with neurologic disorders like Alzheimer’s and Parkinson’s diseases.

**Abstract:**

Angiogenin (ANG), a multifunctional protein known to induce blood vessel formation, is a potential biomarker for cardiovascular diseases; however, whether it is affected by vitamin D supplementation is not known. This interventional study in vitamin D-deficient Saudi adults was designed to investigate it. A total of 100 vitamin D-deficient Saudi adults aged 30–50 years were randomly selected to undergo 6-month vitamin D supplementation. Circulating levels of fasting glucose, lipids, vitamin D, apolipoproteins (AI, AII, B, CI, CII, CIII, E, and H), and ANG were measured using commercially available assays at baseline and after six months. Overall, vitamin D levels increased significantly post intervention. With this, levels of apo-CIII and apo-E significantly increased (*p*-values of 0.001 and 0.009, respectively) with a significant parallel decrease in apo-B (*p* = 0.003). ANG levels were significantly positively associated with most apolipoproteins and inversely correlated with HDL-cholesterol. Post intervention, the changes in ANG levels were positively correlated with apo-E (r = 0.32; *p* < 0.01 in all subjects and r = 0.40; *p* < 0.05 in males). Vitamin D supplementation may modestly affect ANG levels. The association observed between ANG and apo-E is worthy of further investigation since both biomarkers have been linked to neurodegenerative disorders.

## 1. Introduction

Angiogenin (ANG), a ribonuclease discovered by Vallee and colleagues at Harvard in 1985, has a variety of roles. ANG, like all identified ribonucleases (RNases), is represented by a single exon in the core of the RNase gene cluster. When the ANG protein-containing signal peptide is cut, it enhances their secretion [1]. ANG has a promoter or a live-specific promoter responsible for ANG expression, and the promoter is regulated by mRNA transcription. ANG has low ribonucleolytic activity and in vivo does not cleave DNA. The substrate secondary structure determined the specificity of ANG. ANG cuts the three sides of the cytidine monophosphate, or uridine monophosphate [2].

The angiogenic process is strongly controlled in fetal development, repairing injured blood vessels and the SSfemale reproductive system in normal conditions [3]. In recent studies, different roles of calcitriol on angiogenesis depend on the type of the cells, cell condition, and type of chemokines released from the tissue. Vitamin D has anti-angiogenesis effects in the abnormal cell with elevated angiogenesis, such as vascular injury, by lowering the activation, proliferation, and migration [4], whereas, in normal conditions, vitamin D enhances angiogenesis by promoting vascular endothelial growth factor (VEGF) isoforms expression [5]. In various cancer cell lines, vitamin D analogues and vitamin D were found to lower the transcription of VEGF. When Alzheimer’s disease (AD) patients were compared to control people, their serum ANG levels decreased but their VEGF levels increased. Brain function appeared positively related to levels of angiogenin in the serum. Such results can include angiogenin in AD pathogenesis. In the angiogenin gene, the promoter contains vitamin D response elements. Taken together, ANG in AD can be affected by vitamin D [6,7].

Low vitamin D is a global public health issue and Saudi Arabia is not an exception [8,9]. The variables that have been linked to vitamin D deficiency are mostly lifestyle factors, such as morning sleep, daytime behaviors, weather, and job environment [10]. The deficiency of vitamin D showed a positive correlation with age, ethnic location, and seasonal collection of blood samples [11]. In recent years, awareness around the deficiency of vitamin D as well as its therapeutic actions was highlighted by a number of reports. The cardiometabolic effects of vitamin D supplementation have been positively correlated with high density lipoproteins (HDL-cholesterol), while an inverse correlation with low density lipoproteins (LDL-cholesterol, triglycerides) have been found [12,13]. A recent non-linear Mendelian randomization analyses suggests that vitamin D deficiency can increase the risk of cardiovascular diseases [14]. A nested case-control study of 20,025 patients suggests a lower risk of myocardial infarction in individuals maintaining vitamin D levels of ≥75 nmol/L [15].

Due to its immunomodulatory activity, prevention of inflammation, and encouragement of angiogenesis, vitamin D supplementation has also been proposed for medical use in vascular diseases [16]. The protective effect of vitamin D status correction in endothelial cell function has been linked to decreased incidences of thromboembolism [17]. ANG is known to induce blood vessel formation and is a potential biomarker for cardiovascular diseases [18,19,20]. However, limited data is available as to whether vitamin D supplementation affects its circulating levels. This interventional study was thus designed to determine whether vitamin D status correction modulates ANG levels and whether ANG is associated with other known cardiometabolic indices like lipids and apolipoproteins.

## 2. Materials and Methods

### 2.1. Subjects, Vitamin D Supplementation and Clinical Assessment

The cohort used in the present study was taken from the Chair for Biomarkers of Chronic Diseases (CBCD) database of the Biochemistry Department, College of Science, King Saud University (KSU) in Riyadh, Saudi Arabia. For this study, the inclusion criteria were vitamin D-deficient healthy Saudi subjects at baseline (serum level of 25(OH)D < 50 nmol/L) and a total of 100 out of 199 from vitamin D supplementation study were randomly included. The intervention study was approved by Institutional Review Board (IRB) (File# E-15-1667), the Ethics Committee of College of Science, King Saud University (KSU), Riyadh. All clinical indices of subjects were registered, and subjects with cancer, cardiac disease, liver and renal dysfunction, thyroid dysfunction were excluded from the study. To reduce the effect of drugs or any other supplements provided in the present study, also subjects which follow a vitamin D supplementation therapy prior the enrolment were excluded. Vitamin D supplements were provided to all participants as an intervention. Oral 50,000 IU cholecalciferol was given weekly for the first two months, then twice a month for the next two months, followed by daily 1000 IU for the last two months (Synergy Pharma, Dubai, UAE).

Anthropometric and clinical measurements were collected at baseline and after six months. Anthropometrics measurements included height (m), weight (kg), waist (cm), hip circumference (cm), and systolic and diastolic blood pressure (mmHg). Body mass index (BMI) was calculated as weight (kg) divided by height in squared meters. The waist–hip ratio (WHR) was calculated as the quotient between waist and hip circumferences.

### 2.2. Biochemical Measurements

Lipid profile and glucose were measured routinely using a Konelab analyzer (Thermo-Fisher Scientific, Vantaa, Finland). Serum triglycerides ≥ 1.7 mmol/L was considered high [21]. Low HDL-cholesterol was defined as < 1.03 mmol/L in men and < 1.3 mmol/L in women [21,22]. Serum 25 (OH) vitamin D was determined using the LIAISON XL automated quantitative analyzer (DiaSorin, Saluggia, Italy). It uses an advanced chemiluminescence technique with magnetic microparticle separation to achieve the best sensitivity and accuracy of the assay. Vitamin D deficiency was classified 25 (OH) D <50 nmol/L [23]. The apolipoproteins were quantified using MILLIPEX^®^ MAP Human Apolipoprotein Magnetic Bead Panel in Luminex multiplex (Luminexcorp, Austin, TX, USA). Luminex^®^ uses special methods for two fluorescent colors to dye beads internally. A solid-phase assay (Quantikine^®^, R&D Systems, MN, USA) was used to measure ANG levels based on the quantitative sandwich enzyme immunoassay according to the manufacturer’s instructions.

### 2.3. Data Analysis

G*power calculator was used for sample size determination. Using repeated measures analysis, the observed effect size was 0.26 for a total sample size of 100, and the actual observed power was 0.85. Data were analyzed using SPSS (version 21, Armonk, NY, IBM). Categorical variables were presented as frequencies (%). Continuous data were presented as mean ± standard deviation (SD) for variables following Gaussian variables, and non-Gaussian variables were presented in median (1st and 3rd) percentiles. All continuous variables were checked for normality using the Kolmogorov–Smirnov test if not normal, and non-Gaussian variables were log-transformed prior to parametric analysis. Categorical variables were compared using Chi-square tests. Independent Student’s *t*-test was used to determine differences between groups at baseline, and paired *t*-test was used to check mean differences between baseline and follow-up visits. Mann–Whitney U test and Wilcoxon tests were used for non-Gaussian variables whenever applicable. Correlations between variables were done using Spearman’s and Pearson’s correlation analysis. *p*-value < 0.05 was considered statistically significant. All figures were plotted in MS Excel.

## 3. Results

### 3.1. Changes in Biochemical Characteristics in All Subjects Post Intervention

A total of 100 (men 54/women 46) were included in this study with mean age 40.8 ± 9.9 (mean ± S.D). The mean BMI of the subjects was 29.6 ± 5.2 kg/m^2^. Post intervention, the changes in all biochemical characteristics with respect to the baseline values are displayed in Table 1. All subjects were vitamin D-deficient (<50 nmol/L) at baseline, and with vitamin D supplementation, there was a significant improvement in circulating levels of vitamin D (55.2 ± 19.3 follow up vs. 36.2 ± 10.4 nmol/L at baseline, *p*-value (<0.001)). Over the 6-month intervention, most of the metabolic parameters, glucose, triglycerides, HDL-cholesterol, total cholesterol, apo-CI, apo-H, apo-Al, apo-AII, apo-CII, and ANG remained the same except for apo-CIII and apo-E, which showed significant improvements. Changes in different apolipoproteins from baseline to end of study in all subjects reveals a significant average increase of 62.4 µg/mL in apolipoproteins CIII (*p* =0.001) and an average increase of 4.8 µg/mL in APO-E (*p* = 0.009). In contrast, there was a significant mean decrease of 1.73 mg/mL in APO-B post intervention (*p* = 0.003).

### 3.2. Change in Biochemical Characteristics Post Intervention According to Gender

Post-intervention differences according to gender was presented in Table 2. No statistically significant differences were observed in glucose, total cholesterol, HDL-cholesterol, and other clinical characteristics between the males and females at baseline. Males had significantly higher waist–hip ratio and vitamin D levels at baseline compared to females (*p*-values < 0.001 and 0.006, respectively). Moreover, apolipoproteins AII and CII levels were higher in males compared to females at baseline. In both genders, post-intervention 25(OH)D levels significantly increased as expected (*p* < 0.001 for both). Parallel to this, in males, a significant increase in apolipoproteins CII, CIII, and E was observed (*p*-values of 0.02, <0.001, and 0.002 respectively), and in females, a statistically significant decrease in apolipoprotein B was observed (*p* = 0.02). Apo-E in males at baseline were 15.9 (7.5–23.3) and after six months’ supplementation with vitamin D increased significantly to 25.6 (10.5–39.4) (*p* = 0.002). In females, apo-E increased at follow up to 16.6 (2.8–29.0) from 13.8 (2.8–27.5) (*p* = 0.91) at baseline. There was no statistically significant difference in the levels of circulating ANG post intervention in both genders.

**Figure 1:**
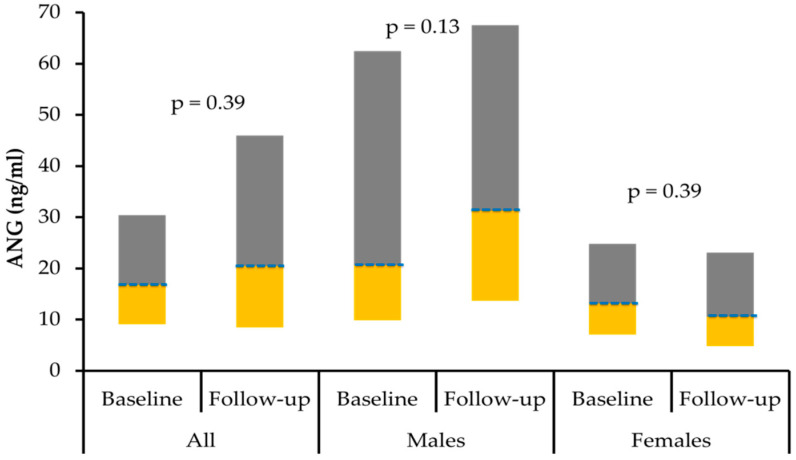
Box-plot representing the median (Q1 and Q3) levels of ANG (ng/mL) for all subjects, males and females.

**Figure 2:**
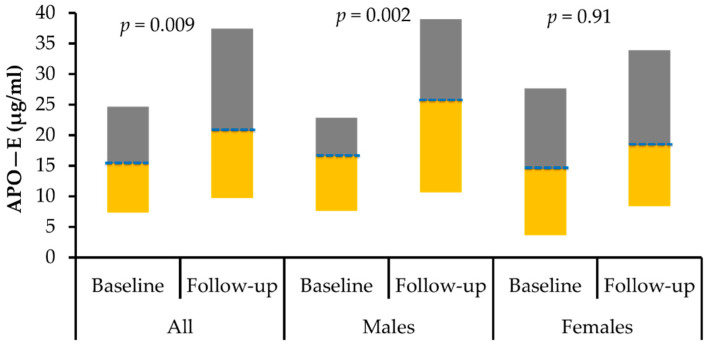
Box-plot representing the median (Q1 and Q3) levels of apolipoprotein E (µg/mL) for all subjects, males and females.

The circulating levels of ANG and Apo-E pre- and post-intervention in both genders were plotted in Figure 1 and Figure 2 respectively.

### 3.3. Correlation of Circulating Levels of ANG with Other Measured Parameters

The bivariate correlation analysis of circulating ANG levels with other measured parameters was displayed in Table 3. In all subjects at baseline, ANG correlate negatively with age, BMI, WHR, HDL-cholesterol, triglyceride, and apo-CI significant. In males, ANG correlated inversely and significantly with glucose and HDL-cholesterol at baseline. There was a significant positive association of ANG with most of the apolipoproteins except apolipoproteins C1 and H. Glucose showed a significant inverse correlation with ANG in males at baseline and in females, post intervention. Interestingly, with vitamin D, ANG showed a positive correlation with vitamin D at baseline, and after vitamin D supplementation, the correlation between ANG and vitamin D changed to negative.

### 3.4. Association of Change in ANG Levels Post Intervention with Other Parameters

The association analysis of the difference (end of study minus baseline values) and delta change (Δ = difference/baseline values) in ANG levels post intervention with other measured parameters was tabulated in Table 4. A significant positive association was seen between the difference levels in ANG and apo-E (r = 0.26, *p* < 0.05) when all subjects were considered. Similarly, the delta change values also showed an association between ANG and apo-E in all subjects (r = 0.32, *p* < 0.01) and in males (r = 0.40, *p* < 0.05).

**Figure 3:**
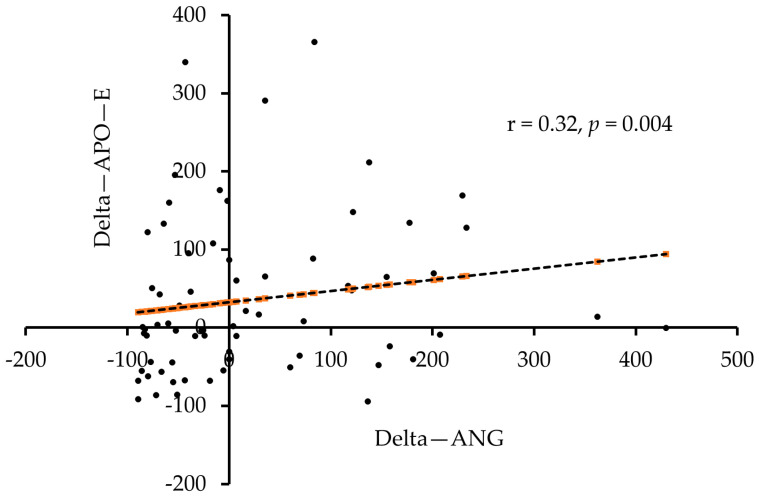
Association between delta change values post intervention in ANG and apo-E in all subjects.

**Figure 4:**
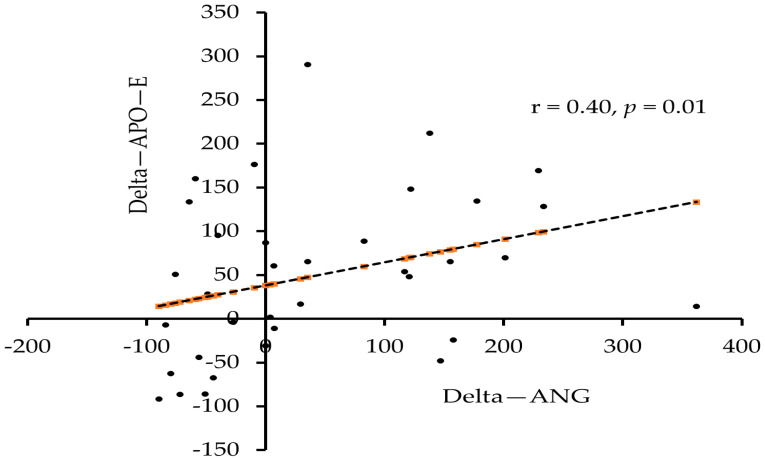
Association between delta change values post intervention in ANG and apo-E in males.

Scatter plots depicting the association between delta change values in ANG and apo-E levels post intervention in all subjects and in males were presented in Figure 3 and Figure 4, respectively.

## 4. Discussion

ANG is a multifunctional protein known to induce blood vessel formation and is a potential biomarker for cardiac deterioration [20] and several types of cancers [24,25,26]. Within this context, we studied the effects of improving vitamin D status in circulating ANG and whether it is associated with other known cardiometabolic indices in vitamin D-deficient adults. This interventional study suggests that vitamin D supplementation may modestly affect ANG levels. To our knowledge, assessing the impact of vitamin D supplementation on circulating ANG has not been conducted previously. Furthermore, this study suggests a sex dimorphic effect on these changes in ANG levels. In addition, this study examined the correlations between ANG levels and metabolic parameters pre- and post-vitamin D supplementation.

ANG has been recently observed to confer protection against pro-inflammatory cytokines in β pancreatic cells and associated with neurodegenerative diseases. In this study, ANG levels modestly increased following correction of vitamin D status as a positive correlation between ANG and vitamin D, especially in females, was observed. This positive correlation between vitamin D and ANG is supported by earlier studies. A study on diabetic foot ulcer patients suggests that vitamin D3 augments proangiogenic factors such as ANG [27]. Lagishetty and his colleagues reported a significant positive correlation between ANG protein and vitamin D [6]. However, there are contrasting results [28] suggesting that vitamin D3 inhibits production of ANG in Human Annulus Cells in vitro. In other studies, hypovitaminosis D was associated with a reduction in serum ANG [7]. The positive association of vitamin D and ANG could be due to an indirect effect through the ANG gene, which contains putative vitamin D response elements in the promoter [6]. In animal models also, vitamin D showed a decreased expression of ANG protein resulting in the higher invasion of colon epithelium by bacteria suggesting a role of vitamin D in the downregulation of tissue inflammation in inflammatory bowel diseases [29,30].

Dyslipidemia, characterized by low levels of HDL-cholesterol (high-density lipoprotein), is a known risk factor of cardiovascular diseases (CVD) [31]. HDL-cholesterol, also known as good cholesterol, transports fats in the body and is a powerful antioxidant [32]. The findings in this study about a negative correlation between circulating levels of ANG and HDL-cholesterol are supported by studies like done by Dworacka and his colleagues [33]. In addition, in a study of individuals with chronic heart failure, correlations between angiogenin levels and cardiac risk factors revealed that ANG was inversely associated with HDL cholesterol [34]. These results suggest that ANG may be useful as an indicator for the progression of vascular disease. In vascular diseases like CVDs, the process of tissue repairing can induce secretion of angiogenic factors like ANG, which may likely be the reason for this inverse correlation with cardio-protective markers like HDL-cholesterol [35].

Apolipoproteins and their association with ANG have been less investigated. In this interventional study, we found that the circulating levels of apolipoproteins AII, B, CII, CIII, and E were positively associated with ANG post intervention. Apo B is mostly generated in the liver, and elevated apo-B levels are linked to an increased risk of coronary heart disease. Apo CII is a lipoprotein lipase (LPL) cofactor, the enzyme that hydrolyzes triglycerides, and apo-CII deficiency stimulates hypertriglyceridemia, an independent atherosclerosis risk factor. Similarly, apo-E is a powerful modulator of the cholesterol efflux process and the formation of HDL particles, both of which promote anti-inflammatory activity [36]. One of the main findings in this study is the changes in ANG were positively associated with apo-E in all participants and males in particular. The apolipoprotein E (apo-E) is a key regulator of lipid metabolism and represents a risk factor for cardiovascular diseases and Alzheimer’s disease [37]. ANG has thus the potential of being a serum biomarker for cancers and cardiovascular diseases [19]. Findings of the present study indicate that ANG and apolipoprotein E are differentially expressed in males and females post vitamin D supplementation and that the extra-skeletal effects of vitamin D correction may benefit males more than females.

Apolipoprotein A-I (Apo A-I) is the main protein portion of high-density lipoprotein (HDL), and it has anti-atherosclerotic activity [38]. Under stressful situations like pathological conditions, apo-AI has been shown to correlate adversely with ANG. Previously, it is well known that under conditions of stress, cells that express mutant apo-A1, ANG expression becomes reduced. Apo-AI clearly induces apoptosis by lowering angiogenin production in order to reduce anti-stress function. This indicates that apo-AI not only controls cholesterol homeostasis in the brain, but also has a role in protecting the brain from injury and stress [39]. The current study contrastingly showed positive correlations between the level of ANG and apo-AI at the baseline, probably reflecting the absence of pathological conditions in the enrolled patients.

Similar to the contrasting association of Apo-A1 and ANG found in this study from the present literature, a contrasting negative association between ANG and glucose, especially in males, was observed in this study. A study done by Patel et al. found a positive association of ANG with plasma glucose [21]. A high concentration of glucose, e.g., in models of Diabetic Retinopathy, has been proposed to induce abnormalities in endothelial cells [40]. A large number of such studies in which this positive association was observed were done in healthy subjects. Glucose intake affects systemic inflammation and oxidative stress; however, this happens through a complex inflammatory response mechanism which needs to be further evaluated [41,42].

In our study, no improvement was observed in glucose levels after six-month of vitamin D supplementation. In contrast, studies suggested a direct positive and beneficial effect on glucose homeostasis with vitamin D supplementation in patients with diabetes [43]. Varied responses to vitamin D supplementation have been reported and linked with the potential role of vitamin D in enhancing the metabolism of glucose [44]. However, in this study, the reason for no improvement in glucose levels may be due to the fact that our subjects at baseline were healthy and had normal glucose levels.

Our study showed a significant decrease in apoB with vitamin D supplementation. ApoB has been associated with atherogenic lipid particles mainly low density lipoprotein [45] and the negative correlation between the two suggests atheroprotective properties of vitamin D supplementation. Some earlier studies, however, reported no association between apoB with vitamin D supplementation [46]. Some others reported a marked increase in apoB circulating levels [47]. These conflicting results may be due to different methods used, sex differences, and variations in the hormonal response of target tissue contributing to variations in lipid control and gene activation/inactivation.

This interventional study found a significant elevation in levels of apo-CIII and apo-E after total adjustment of vitamin D levels which is in line with interventional studies that found a significant link between apo-E and vitamin D intake from food sources [48].The rise of these apolipoproteins in this interventional study suggests the positive modulation of vitamin D on the cardiometabolic factors. The marked increase in apo-CIII with vitamin D supplementation because of the atherogenic properties of apo-CIII appears controversial, but it must be looked at considering the fact that apo-CIII is readily exchangeable between triglyceride-rich lipoprotein and HDL systems [23]. Interestingly, research among people with arterial hypertension showed a significant rise in apo-CIII and apoE [32] supporting our results.

The authors acknowledge some limitations in this study. The first is the lack of a control arm, which may have provided an intriguing viewpoint on the vitamin D intervention. Another limitation is the sample size, and the authors admit that a larger investigation would be required to reinforce the findings. Furthermore, factors impacting vitamin D status such as season, sunlight exposure, and outdoor activity were not included in this study. Nevertheless, the study presented results of clinical importance suggesting modulation of ANG levels by vitamin D supplementation, and future such studies would be required to improve our understanding of ANG and association with vitamin D.

## 5. Conclusions

The present study suggests that increasing 25(OH)D levels through supplementation may modestly affect ANG levels. Furthermore, the association between ANG and apo-E observed in adult Saudi males is worthy of further investigation since both biomarkers have been linked to neurodegenerative disorders.

## Figures and Tables

**Table 1:** Changes in biochemical parameters after Vitamin D Supplementation.

Parameters	All Subjects		
	Baseline	Follow-Up	*p*-Value
N (Males/Females)	100 (54/46)		
Age (years)	40.8 ± 9.9		
BMI (kg/m^2^)	29.6 ± 5.2		
WHR	0.93 ± 0.1		
Systolic BP (mmHg)	127.1 ± 13.3		
Diastolic BP (mmHg)	79.5 ± 9.3		
Glucose (mmol/L)	5.75 ± 1.2	5.79 ± 1.1	0.82
Total Cholesterol (mmol/L)	4.9 ± 1.0	5.1 ± 1.2	0.24
HDL-Cholesterol (mmol/L)	1.01 ± 0.4	1.09 ± 0.4	0.12
Triglycerides (mmol/L)	1.5 (1.1, 2.2)	1.7 (1.1, 2.3)	0.22
25(OH)D (nmol/L)	36.2 ± 10.4	55.2 ± 19.3	<0.001
Apo-CI (µg/mL)	35.3 (28.2, 48.3)	35.5 (29.8, 52.2)	0.32
Apo-H (µg/mL)	412.3 ± 139.2	378.8 ± 157.8	0.15
Apo-Al (mg/mL)	19.78 (5.7, 7.5)	18.82 (7.3, 61.3)	0.64
Apo-CIII (µg/mL)	137.8 (66.9, 211.8)	200.2 (63.5, 358.9)	0.001
Apo-E (µg/mL)	14.8 (7.3, 24.3)	19.6 (8.7, 36.6)	0.009
Apo-AII (mg/mL)	0.93 (0.6, 0.9)	0.91 (0.4, 1.4)	0.36
Apo-B (mg/mL)	7.99 (5.2, 11.3)	6.26 (3.5, 9.7)	0.003
Apo-CII (µg/mL)	115.4 (69.4, 209.6)	136.1 (71.9, 219.6)	0.26
ANG (ng/mL)	16.5 (8.9, 27.8)	19.1 (7.9, 36.9)	0.39

Note: Data Presented as Mean ± SD and Median (1st Q, 3rd Q) for Gaussian and Non-Gaussian variables. *p*-value was calculated for differences between baseline and 6-month data, and *p* < 0.05 was considered as significant.

**Table 2:** Changes in clinical characteristics post intervention in males and females.

Parameters	Males			Females		
	Baseline	Follow-Up	*p*-Value	Baseline	Follow-Up	*p*-Value
N (M/F)	54	54		46	46	
Age (years)	41.9 ± 9.8			39.5 ± 9.9		
BMI (kg/m^2^)	28.8 ± 4.7			30.6 ± 5.5		
WHR	0.97 ± 0.1			0.88 ± 0.1		
Systolic BP (mmHg)	129.9 ± 11.1			123.5 ± 14.9		
Diastolic BP (mmHg)	80.6 ± 8.0			78.1 ± 10.7		
Glucose (mmol/L)	6.02 ± 1.2	6.03 ± 1.3	0.97	5.5 ± 1.3	5.6 ± 0.9	0.74
T. Chol. (mmol/L)	4.9 ± 1.0	5.1 ± 1.1	0.34	4.9 ± 0.9	5.0 ± 1.2	0.49
HDL-C (mmol/L)	0.98 ± 0.4	1.04 ± 0.4	0.22	1.05 ± 0.4	1.14 ± 0.4	0.29
Trig.(mmol/L)	1.50 (1.1, 2.4)	1.60 (1.2, 2.0)	0.45	1.4 (1.1, 1.9)	1.8 (1.1, 2.4)	0.26
25(OH) D (nmol/L)	38.8 ± 9.6	55.1 ± 12.8	<0.001	33.1 ± 10.6	55.2 ± 24.9	<0.001
Apo-CI (µg/mL)	32.6 (23.8, 36.5)	31.8 (26.6, 35.5)	0.86	44.4 (32.2, 61.1)	44.8 (36.6, 75.6)	0.15
Apo-H (µg/mL)	396.7 ± 131.4	374.2 ± 159.9	0.53	424.9 ± 145.4	382.5 ± 157.8	0.18
Apo-Al (mg/mL)	19.37 (6.3, 49.2)	18.28 (7.2, 26.6)	0.24	23.40 (4.2, 212.3)	22.98 (6.9, 161.9)	0.09
Apo-CIII (µg/mL)	139.1 (70.8, 280.9)	256.6 (124.1, 451.4)	<0.001	120.9 (57.9, 178.4)	148.1 (24.5, 271.7)	0.53
Apo-E (µg/mL)	15.9 (7.5, 23.3)	25.6 (10.5, 39.4)	0.002	13.8 (2.8, 27.5)	16.6 (2.8, 29.0)	0.91
Apo-AII (mg/mL)	1.23 (0.8, 1.6)	1.25 (0.8, 1.7)	0.55	0.72 (0.4, 1.2)	0.62 (0.3, 0.9)	0.07
Apo-B (mg/mL)	8.12 (6.4, 12.3)	8.46 (4.5, 10.0)	0.08	7.86 (3.8, 9.5)	4.58 (1.8, 8.0)	0.02
Apo-CII (µg/mL)	153.3 (90.3, 223.1)	194.8 (112.1, 257.4)	0.02	100.8 (56.9, 166.1)	101.9 (28.3, 142.9)	0.24
ANG (ng/mL)	19.1 (9.4, 38.4)	27.5 (11.6, 51.0)	0.13	13.3 (7.2, 25.3)	10.3 (4.4, 22.9)	0.39

Note: Data Presented as Mean ± SD and Median (1st Q, 3rdQ) for Gaussian and Non-Gaussian variables, respectively. *p*-value represents the statistical significance for the differences between pre- and post-intervention. *p* < 0.05 is considered significant for all tests.

**Table 3:** Association of circulating ANG with other measured parameters.

Parameters	Baseline			Follow-Up		
	All	Males	Females	All	Males	Females
N (M/F)	100	54	46	100	54	46
Age	−0.21 *	−0.11	−0.34 *	−0.04	−0.03	0.15
BMI	−0.24 *	−0.26	−0.18	−0.29 **	−0.16	−0.24
WHR	−0.26 *	−0.43 **	−0.36 *	0.08	−0.06	−0.31
Systolic BP	0.02	0.03	−0.10	−0.03	−0.02	−0.30
Diastolic BP	−0.06	−0.12	−0.04	0.13	0.18	−0.05
Glucose	−0.20	−0.38 **	−0.15	−0.12	−0.05	−0.36 *
Total-Cholesterol	0.02	0.01	0.10	0.10	0.10	0.10
HDL-Cholesterol	−0.25 *	−0.41 **	−0.06	0.12	0.15	0.25
Triglycerides #	−0.22 *	−0.24	−0.25	−0.13	−0.05	−0.22
Vitamin D	0.26 *	0.08	0.43 **	−0.23 *	−0.18	−0.32 *
Apo-CI #	−0.26 *	−0.15	−0.24	−0.37 **	−0.01	−0.51 **
Apo-H	−0.10	−0.02	−0.01	−0.26 *	−0.36 *	−0.17
Apo-Al #	0.44 **	0.41 **	0.49 **	0.13	−0.10	0.45 **
Apo-CIII #	0.45 **	0.42 **	0.51 **	0.45 **	0.26	0.48 **
Apo-E #	0.54 **	0.60 **	0.51 **	0.49 **	0.35 *	0.56 **
Apo-AII #	0.42 **	0.43 **	0.32 *	0.44 **	0.11	0.58 **
Apo-B #	0.27 *	0.31 *	0.18	0.34 **	0.10	0.47 **
Apo-CII #	0.39 **	0.38 **	0.36 *	0.41 **	0.22	0.37 *

Note: Data presented as correlation coefficient (r); # denotes non-normal variables which were log-transformed before analysis; * denotes significance at 0.05 level; ** denotes significance at 0.01 level.

**Table 4:** Association of difference and delta change (Δ) values post intervention between ANG levels and other measured parameters.

Parameters	Difference Post Intervention (ANG)			Δ Post Intervention (ANG)		
				Δ (All)	Δ (Males)	Δ (Females)
N (M/F)	100	54	46	100	54	46
Glucose	0.10	0.16	0.10	0.10	0.21	0.03
Total-Cholesterol	0.02	0.01	0.02	−0.02	−0.10	0.01
HDL-Cholesterol	−0.10	−0.21	−0.01	−0.06	−0.28	0.05
Triglycerides #	0.06	0.04	0.12	−0.04	−0.10	0.02
Vitamin D	0.03	0.10	−0.10	−0.01	0.16	−0.15
Apo-CI #	0.10	0.14	0.13	0.07	0.17	−0.01
Apo-H	−0.20	−0.25	−0.12	−0.22	−0.26	−0.15
Apo-Al #	−0.11	−0.18	0.04	0.10	0.01	0.20
Apo-CIII #	0.03	0.03	−0.16	0.12	0.14	0.03
Apo-E #	0.26 *	0.24	0.01	0.32 **	0.40 *	0.11
Apo-AII #	0.04	−0.08	0.03	0.20	0.12	0.27
Apo-B #	0.08	0.11	−0.15	0.20	0.19	0.18
Apo-CII #	0.02	0.05	−0.23	0.16	0.16	0.10

Note: Data presented as correlation coefficient (r); # denotes non-normal variables which were log-transformed before analysis; * denotes significance at 0.05 level; ** denotes significance at 0.01 level; Δ represents the delta change values.

## Data Availability

The data used to generate the results of this study are available from the corresponding author on reasonable request taking into account the patient data policy of our institution.
